# Efficacy and safety of biosimilar Peg-filgrastim after autologous stem cell transplant in myeloma and lymphoma patients: a comparative study with biosimilar Filgrastim, Lenograstim, and originator Peg-filgrastim

**DOI:** 10.1007/s00277-023-05604-9

**Published:** 2024-01-08

**Authors:** Francesco Marchesi, Irene Terrenato, Elena Papa, Martina Tomassi, Paolo Falcucci, Svitlana Gumenyuk, Francesca Palombi, Francesco Pisani, Daniela Renzi, Atelda Romano, Antonio Spadea, Giulia Regazzo, Maria Giulia Rizzo, Mafalda De Rienzo, Claudio Ripellino, Simona Sgromo, Caterina Viggiani, Eleonora Ponte, Ramy Kayal, Iole Cordone, Maria Laura Foddai, Andrea Mengarelli

**Affiliations:** 1 Hematology Unit, IRCCS Regina Elena National Cancer Institute, Via Elio Chianesi 55, 00144 Rome, Italy; 2 Department of Research, Advanced Diagnostics and Technological Innovation, Clinical Trial Center, Biostatistics and Bioinformatics Unit, IRCCS Regina Elena National Cancer Institute, Rome, Italy; 3 Department of Research, Advanced Diagnostics and Technological Innovation, Genomic and Epigenetic Unit, Translational Research Area, IRCCS Regina Elena National Cancer Institute, Rome, Italy; 4 Immuno-Transfusional Medicine Unit, IRCCS Regina Elena National Cancer Institute, Rome, Italy; 5 Health Economics & Real-World Evidence Freelance, Milan, Italy; 6 Leukapheresis and Cellular Therapy Unit, S. Camillo-Forlanini Hospital, Rome, Italy; 7 Radiology Unit, IRCCS Regina Elena National Cancer Institute, Rome, Italy; 8 Clinical Pathology Unit, IRCCS Regina Elena National Cancer Institute, Rome, Italy

**Keywords:** Autologous stem cell transplant, Biosimilars, Granulocyte colony-stimulating factors, Myeloma, Lymphoma, Peg-filgrastim

## Abstract

Data about biosimilar Peg-filgrastim (bioPEG) in autologous stem cell transplant (ASCT) are still scarce. The aim of this study has been to assess efficacy and safety of bioPEG among lymphoma and myeloma patients undergoing ASCT, comparing these data with historical controls receiving other G-CSFs. Furthermore, an economic evaluation has been included to estimate the savings by using bioPEG. This is a prospective cohort study comparing lymphoma and myeloma patients undergoing ASCT and receiving bioPEG (*n* = 73) with three historical consecutive cohorts collected retrospectively who received other G-CSFs (Lenograstim — Leno — *n* = 101, biosimilar Filgrastim — bioFIL *n* = 392, and originator Peg-filgrastim — oriPEG *n* = 60). We observed a significantly shorter time to neutrophils and platelet engraftment (*p* < 0.001) in patients treated with bioPEG and oriPEG. Moreover, patients who received bioPEG showed a shorter hospitalization time (*p* < 0.001) and a lower transfusion need (*p* < 0.001). We did not observe any significant difference in terms of transplant-related mortality, mucositis, and diarrhea among the four groups. No serious adverse events were associated with bioPEG. Similar data were obtained after running a stratified analysis for lymphomas and myeloma separately conducted by using a propensity score matching. The average total cost per patient of bioPEG was € 18218.9 compared to € 23707.8, € 20677.3 and € 19754.9 of Leno, oriPEG, and bioFIL, respectively. In conclusion, bioPEG seems to be as effective as the originator and more effective than short-acting G-CSFs in terms of post-transplant engraftment in myeloma and lymphoma patients undergoing ASCT. Moreover, bioPEG was cost-effective when compared with the other G-CSFs.

## Background

Autologous hematopoietic stem cell transplant (ASCT) is performed after administration of high dose chemotherapy, called conditioning regimen, typically determining a detrimental effect on bone marrow and causing severe neutropenia, thrombocytopenia, and anemia [1]. The most widely used conditioning regimes around the globe are MEL200 (melphalan 200 mg/sqm) for multiple myeloma patients, BEAM (carmustine, etoposide, cytarabine, and melphalan) or BEAC (carmustine, etoposide, cytarabine, and cyclophosphamide) for lymphoma patients and BU-CY2 (busulfan and cyclophosphamide) for acute leukemia patients [2]. Nowadays, after the approval of novel cellular therapy approaches (i.e., CAR-T), the right placement of ASCT in the therapeutic algorithm of some hematologic malignancies is under debate, but so far still remains the standard of care in several settings [2]. ASCT can be associated with several side effects and potentially death (transplant-related mortality, TRM) mainly due to infections and hemorrhages. However, in the last years, TRM has constantly dropped given the improvement of our knowledge of supportive measures [3]. In particular, the administration of granulocyte colony stimulating factors (G-CSF) after stem cell infusion permitted a faster neutrophil number recovery and consequent drop in febrile neutropenia events, infection occurrence, antibiotic use, and hospital stay days [4]. Several G-CSF have been so far approved for febrile neutropenia prophylaxis in patients undergoing high-dose chemotherapy, divided in short-acting and long-acting. The first ones are characterized by a daily administration until neutrophil engraftment and are Filgrastim, biosimilar Filgrastim, and Lenograstim. On the contrary, long-acting G-CSFs (Peg-filgrastim, biosimilar Peg-Filgrastim, and Lipefilgrastim) are given as one-shot post-chemo administration as a consequence of their long lasting action [5]. Since the implementation of a biosimilar approval pathway in 2005, several biosimilars including somatotropins, filgrastim, epoietins, and infliximab have been approved in Europe, on the basis of demonstrating comparable quality, safety, and efficacy to the originator products [6]. Biosimilars are biological drugs whose target and mechanisms of action are the same as those of an originator biological drug. A “biosimilar” is correctly defined as a drug that has been approved in highly regulated markets and that meets stringent criteria of quality and comparability to its respective originator biologic product. The development of biosimilars is achieved by applying the same evidence-based regulatory standards as originator products, where cost limitations do not reflect lower quality, efficacy, and safety, or worsening of patient outcomes [6, 7]. Biosimilar G-CSFs are substantially identical to the originators and the only small differences in the microheterogeneity pattern of the molecule do not translate in meaningful clinical differences in terms of both efficacy and safety [8]. The key driver for uptake of biosimilars is cost reduction relative to the originator biologics; in fact, biosimilars are at least 15–45% less expensive than the originator biologics [9]. A variety of incentives and policies have been implemented in Europe to promote market access and uptake of biosimilars. The main reason for this favorable market is that countries wish to capture the savings resulting from the lower cost of biosimilars in an era of limited healthcare budgets, increasing burden of life-threatening diseases, earlier detection of diseases, and increasing aging population [10]. As a consequence, economic assessments that offer a comprehensive estimate of savings represent important decision-making tools for the payer. In 2019, the Food and Drug Administration (FDA) approved the first biosimilar Peg-filgrastim with the same indications of its originator. Even though the use of biosimilar Peg-filgrastim is progressively increasing in oncohematology field, there are still few data about its efficacy and safety in patients undergoing ASCT and allogeneic hematopoietic stem cell transplant. As for ASCT, a recent study from an Italian transplant center have explored the use of biosimilar Peg-filgrastim among multiple myeloma patients undergoing ASCT suggesting a substantial superimposable efficacy and safety compared to other G-CSFs (originator Peg-filgrastim and biosimilar short-acting filgrastim) [11]. The aim of this study has been to assess the efficacy and the safety of biosimilar Peg-Filgrastim among a lymphoma and myeloma patient population undergoing ASCT, comparing these data with those derived by our historical control groups of patients receiving other G-CSFs for febrile neutropenia prophylaxis (i.e., biosimilar Filgrastim, originator Peg-filgrastim, and Lenograstim). Furthermore, an economic evaluation has been included to estimate the savings associated with the use of biosimilar Peg-Filgrastim compared to other therapeutic options.

## Methods

### Study design and patients

This is a prospective cohort study comparing lymphoma and myeloma patients undergoing ASCT at Hematology Unit of Regina Elena National Cancer Institute and receiving biosimilar Peg-filgrastim with three historical consecutive patient cohorts collected retrospectively who received other G-CSF formulations at the same institution. The main study patient cohort included 73 patients affected by multiple myeloma or lymphoma who consecutively underwent ASCT at our institution between June 2021 and May 2023. Inclusion criteria were age above 18 years, eligibility for ASCT and availability of a post-transplant 3 month-long follow-up for collecting data. Patients in all disease phases (i.e., complete remission — CR, very good partial remission — VGPR, partial remission — PR, stable disease — SD, or progressive disease — PD) were included.

### Procedures

For all patients, we collected the following data: sex, age, diagnosis, induction treatment before ASCT, and number of chemotherapeutic lines, disease status at ASCT, number of CD34 +  × 10^6^/kg collected and actually infused, conditioning regimens, days to neutrophil and platelet engraftment, febrile and infectious episodes, other side effects, antibiotic and transfusion needing, days of hospitalization, and TRM. In all enrolled patients, a single administration of Peg-filgrastim at 6 mg was subcutaneously given at day 3 after stem cell infusion. This cohort of patients was compared with three historical cohorts (Table 1): (a) 392 consecutive adult patients treated with biosimilar Filgrastim at dosage of 5 mcg/kg daily given from day 3 after infusion until neutrophil engraftment from March 2013 to May 2021; (b) 101 consecutive adult patients treated with Lenograstim at a dosage of 5 mcg/kg daily given from day 3 after infusion until neutrophil engraftment from January 2009 to February 2013; (c) 60 consecutive adult patients treated with originator Peg-filgrastim at a dosage of 6 mg single dose at day 3 after infusion from March 2006 to December 2008. All multiple myeloma patients younger than 65 years received a MEL200 conditioning regimen, whereas patients aged above 65 years received a reduced intensity conditioning regimen with melphalan 140 mg/sq m (MEL140). All but 60 lymphoma patients received BEAM conditioning regimen; those 60 patients received FEAM conditioning regimen between September 2011 and August 2015 due to difficult supply of carmustine.

**Table 1:** Baseline features of patients according to the received G-CSF formulation

Parameter	Biosimilar Peg-filgrastim, *N* = 73	Biosimilar Filgrastim *N* = 392	Lenograstim, *N* = 101	Originator Peg-filgrastim, *N* = 60	*p*-value
	*N* (%)	*N* (%)	*N* (%)	*N* (%)	
Sex, M/F, *N* (%)	33/40 (45/55)	24/181 (54/46)	64/37 (63/37)	36/24 (60/40)	0.089*
Median age, years (range)	60 (31–70)	57 (19–72)	55 (19–72)	56 (18–71)	< 0.001**
Diagnosis					0.004*
MM	57 (78)	227 (58)	66 (65)	26 (43)	
NHL/HD	16 (22)	165 (42)	35 (35)	34 (57)	
Median chemotherapy lines prior ASCT (range)	1 (1–2)	1 (1–5)	2 (1–4)	2 (1–4)	< 0.001**
Disease status at ASCT					0.071*
CR	39 (53)	205 (52)	64 (63)	38 (63)	
PR	34 (47)	166 (42)	35 (35)	21 (35)	
SD/PD	-	21 (6)	2 (2)	1 (2)	
Conditioning chemotherapy					0.004*
MEL200/MEL140	57 (78)	227 (58)	66 (65)	26 (43)	
BEAM/FEAM	16 (22)	165 (42)	35 (35)	34 (57)	
Median infused CD34 + cells × 10^6^/kg (range)	4.83 (2.92–10.36)	5.62 (3.37–16.68)	5.53 (3.31–14.50)	5.42 (2.93–14.4)	< 0.001**

Bold values are those statistically significant

*MM* multiple myeloma; *NHL* non-Hodgkin’s lymphoma; *HD* Hodgkin’s disease; *ASCT* autologous stem cell transplantation; *CR* complete remission; *PR* partial remission; *SD* stable disease; *PD* progressive disease; *MEL200* melphalan 200 mg/sq m; *MEL* 140 melphalan 140 mg/sq m; *BEAM* carmustine, etoposide, cytarabine, melphalan; *FEAM* fotemustine, etoposide, cytarabine, melphalan

^*^ Pearson’s chi-square test ** Kruskall-Wallis test

All patients received the same institutional standard procedures. In particular, oral Valacyclovir was given at the dosage of 1 g/day from the day of stem cell infusion to 6 months after transplant for herpes viruses prophylaxis. *Pneumocystiis jirovecii* pneumonia prophylaxis was administered with Trimethoprim/Sulfamethoxazole 1 double strength tablet twice a week from the day of stem cell infusion to 6 months after transplant. No anti-microbial prophylaxis was given. Red blood cell (RBC) and platelet transfusion were administered for hemoglobin level < 8 g/dL and platelet count < 10 × 10^9^/L or in patients with symptomatic anemia or hemorrhagic syndrome. Intravenous hydratation and electrolyte support was given according the good clinical practice and the institutional standard protocols. Febrile neutropenia was homogenously managed according to the institutional protocols in all patients. In particular, empirical broad-spectrum anti-microbial treatment was promptly started at the onset of fever (defined as body temperature ≥ 38 °C in two consecutive determinations) during neutropenia (defined as ANC ≤ 0.5 × 10^9^/L) with a combination of Piperacillin-tazobactam plus Amikacin, following the local protocols and the international guidelines [12, 13]. Hematologic engraftment after ASCT was defined as an absolute neutrophil count upper than 0.5 × 10^9^/L and a platelet count upper than 20 × 10^9^/L in three consecutive checks. Other adverse events were collected and graded according to the Common Toxicity Criteria of the National Cancer Institute.

### Ethical approval

The study was approved by the local institutional review board and ethical committee (approval protocol number: 36/IRE/23–2866) and conducted in accordance to the Helsinki Declaration and the International Conference on Harmonization Guidelines for Good Clinical Practice.

### Study objectives

The primary objective of the study was to evaluate the efficacy in terms of ANC engraftment after stem cell infusion in our patient population receiving a single dose of biosimilar Peg-filgrastim after ASCT. Secondary objectives of the study included platelet engraftment after stem cell infusion, febrile neutropenia, documented infections, and antibiotic use, transfusion and hospitalization days, safety, and TRM. For all these objectives, we also performed a comparison of patients receiving biosimilar Peg-filgrastim with those receiving other G-CSF formulations.

### Statistical analysis

Descriptive statistics were calculated for all variables of interest. Categorical variables were summarized through frequencies and percentage values while continuous variables through median or mean values and their relative variability index (standard deviation or range, respectively). All distributions were tested for normality by Shapiro–Wilk test, and the most suitable test was applied to compare groups: Pearson’s chi-square test or Fisher for categorical variables and the Mann–Whitney test or Kruskall-Wallis test for continuous variables. Bonferroni correction for multiple comparisons was applied, when appropriate. Stratified analyses for lymphoma and myeloma patients were conducted. In this specific sub-group, in order to control for potential confounders that could affect the outcomes of interest when we compared the different G-CSFs, propensity score matching (PSM) was employed to generate two different pair wise groups with balanced distribution of specific baseline features [14]. The dependent variable was the choice of specific G-CSF: biosimilar Peg-filgrastim was considered the experimental drug, while Lenograstim, biosimilar Filgrastim and Peg-filgrastim were considered the control group for each sub-analysis. Patients were matched one-to-one with a tolerance of 0.5 for age at diagnosis, gender, and number of CD34 infused cells. A *p*-value < 0.05 was considered statistically significant. All statistical analyses were carried out with IBM SPSS v 29.0.

### Costs analysis

A cost analysis in terms of average total and saved cost per person between the groups was conducted. G-CSF treatment, intravenous empirical broad-spectrum antibiotic needing (piperacillin-tazobactam 4.5 g every 6 h plus 20 mg/kg/daily for an average duration of 8 days), RBC and PLT transfusions, and days of hospitalization, which clinical and resource utilization data derive from the main study, were considered for the economic evaluation. Treatment costs were calculated considering the lowest ex-factory prices (October 2023); the costs of blood components and related processes, inclusive for all costs incurred for self-donation by the transfusion service, were estimated by using the tariffs of interregional agreement for the compensation of healthcare mobility [15], and the average cost of hospitalization per day was estimated in the 2004 by the Italian Ministry of Economy and Finance [16], and it was revalued to 2022 based on the ISTAT consumer price index (Table 2).

**Table 2:** Unit costs

	Euro (€)
Biosimilar peg-filgrastim 6 mg	390.00 €
Lenograstim 34 MUI/mL *	87.96 €
Originator Peg-filgrastim 6 mg	1000 €
Biosimilar Filgrastim 30 MU *	63.90 €
Piperacillin-Tazobactam 4 + 0.5 g 10 units	83.77 €
Amikacin 500 mg 10 units	18.31 €
Cost of blood components and related processes (RBC)	181.00 €
Cost of blood components and related processes (PLT)	418.00 €
Average cost of hospitalization per day	905.86 €

*RBC* red blood cells, *PLT* platelets

^*^ 1 vial per day was considered for an assumed patient with a median body weight of 70 kg

## Results

### Analysis on the overall population

From June 2021 to May 2023, 73 consecutive patients with multiple myeloma (57) and lymphoma (16) underwent ASCT and received biosimilar Peg-filgrastim after stem cell infusion. Baseline demographic and clinical characteristics of these patients and of the three historical control groups (as above defined) are resumed in Table 1. As shown, patients who received biosimilar Peg-filgrastim were significantly older (*p* < 0.001), more frequently affected by myeloma and then treated with melphalan-based conditioning regimens (*p* = 0.004) and were infused with a significantly lower number of CD34 + cells (*p* < 0.001). On the contrary, no significant differences as for sex distribution and disease status at transplant were observed among the four patient cohorts (*p* = 0.089 and 0.071, respectively). Table 3 shows the post-transplant clinical outcomes by the received G-CSF formulation. We observed a shorter time to neutrophil engraftment in the cohort of patients treated with both biosimilar and originator Peg-filgrastim (*p* < 0.001), with a median time of 10 days among those patients, compared with 11 days achieved in the cohort of patients receiving short-acting G-CSFs, as biosimilar Filgrastim and Lenograstim. The same result was observed for PLT engraftment, significantly faster in Peg-filgrastim groups (biosimilar 11 days and originator 12 days) than in the other two groups (biosimilar Filgrastim 13 days, Lenograstim 14 days; *p* < 0.001). As for the other analyzed parameters, we did observe a similar incidence of febrile neutropenia episodes, microbiologically documented infections and intravenous antibiotic needing among the four patient cohorts (*p* = 0.770, *p* = 0.493, and *p* = 0.770, respectively). In contrast, our data showed a significant lower RBC and platelet transfusion rate in patients receiving both biosimilar and originator Peg-filgrastim, when compared with the other two groups (*p* < 0.001). In addition, a shorter median duration of the hospitalization was observed in the patient cohort treated with biosimilar Peg-filgrastim (19 days; *p* < 0.001). In particular, the advantage was statistically significant when compared with Lenograstim group (*p* = 0.001) and did not reach the statistical significance when compared with the other two groups (originator Peg-filgrastim and biosimilar Filgrastim). Finally, we did not observe any significant difference in terms of TRM among the four groups of patients. No significant differences in terms of mucositis and diarrhea were observed among the four groups of patients. No grade 3–4 adverse events were associated with the biosimilar Peg-filgrastim administration.

**Table 3:** Post-transplant clinical outcomes by the received G-CSF formulation

Results	Biosimilar Peg-filgrastim, *N* = 73	Biosimilar Filgrastim, *N* = 392	Lenograstim, *N* = 101	Originator Peg-filgrastim, *N* = 60	*p*-value
Engraftment					
Median days (range) at ANC > 0.5 × 10^9^/L	10 (9–12)	11 (5–30)	11 (9–29)	10 (8–18)	< 0.001**
Median days (range) PLTs > 20 × 10^9^/L	11 (9–16)	13 (5–120)	14 (10–35)	12 (9–23)	< 0.001**
Median days (range) G-CSF injections		8 (4–26)	9 (4–26)		** < 0.001°**
Febrile neutropenia episodes (%)	34 (47%)	208 (53%)	54 (63%)	32 (53%)	0.770*
Microbiologically documented infections (%)	24 (33%)	153 (39%)	43 (43%)	20 (33%)	0.493*
Intravenous antibiotics needing (%)	34 (47%)	208 (53%)	54 (63%)	32 (53%)	0.770*
Mean number (SD) RBC transfusions	0.3 (1.4)	0.7 (1.9)	0.8 (1.5)	0.4 (0.9)	0.004**
Median number PLT transfusions (range)	1 (0–15)	2 (0–18)	2 (0–12)	1 (0–6)	< 0.001**
Median hospitalization duration, days (range)	19 (14–59)	20 (13–66)	24 (15–68)	21 (6–29)	< 0.001**
TRM (%)	1 (1%)	8 (2%)	4 (4%)	1 (2%)	0.619*

Bold values are those statistically significant

*RBC* red blood cells, *PLT* platelets, *ANC* absolute neutrophilis count, *TRM* transplant-related mortality, *SD* standard deviation

^*^ Pearson’s chi-square test; ** Kruskal–Wallis test

° Mann–Whitney test

### Sub-analysis for lymphoma and myeloma patients

A stratified analysis for lymphoma and myeloma patients only was conducted by using PSM to generate different pair wise groups with balanced distribution of specific baseline features. Setting the tolerance at 0.5 allowed creating different sub-groups in terms of sample size. As shown in Table 4, lymphoma patients who received biosimilar Peg-filgrastim have a shorter median time to neutrophil engraftment than patients receiving Lenograstim (10 vs 11 days; *p* < 0.001) and biosimilar Filgrastim (10 vs 11 days; *p* < 0.001), whereas the better performance was observed in patients treated with originator Peg-filgrastim (9 vs 10 days; *p* = 0.009). As for the other analyzed parameters, we did not observe significant differences among the four patient cohorts, except for a better time to PLT engraftment and a lower needing for RBC transfusions in the biosimilar Peg-filgrastim group compared with biosimilar Filgrastim (*p* = 0.016 and *p* = 0.024, respectively); a lower number of RBC and platelet transfusions and a lower hospitalization time in the biosimilar Peg-filgrastim group compared with Lenograstim (*p* = 0.012, *p* = 0.012, and *p* = 0.022, respectively). Similar results are carried out from myeloma sub-analysis, as shown in Table 5.

**Table 4:** Post-transplant clinical outcomes by the received G-CSF formulation. Pairwise propensity score matching analysis in lymphoma patients

Results	Biosimilar Peg-filgrastim, *N* = 13	Lenograstim, *N* = 13	*p*-value	Biosimilar Peg-filgrastim, *N* = 16	Originator Peg-filgrastim, *N* = 16	*p*-value	Biosimilar Peg-filgrastim, *N* = 16	Biosimilar Filgrastim, *N* = 16	*p*-value
Engraftment									
Median days (range) at ANC > 0.5 × 10^9^/L	10 (9–10)	11 (10–16)	** < 0.001°**	10 (9–11)	9 (8–10)	**0.009°**	10 (9–11)	11 (9–15)	** < 0.001°**
Median days (range) PLTs > 20 × 10^9^/L	12.5 (9–15)	12 (10–26)	0.802°	12 (9–15)	13 (9–17)	0.561°	12 (9–15)	14 (10–45)	**0.016°**
Febrile neutropenia episodes (%)	10 (77%)	10 (77%)	1.000**	12 (75%)	13 (81%)	1.000**	12 (75%)	9 (56%)	0.458**
Microbiologically documented infections (%)	5 (38%)	8 (62%)	0.239*	7 (44%)	6 (37%)	0.719*	7 (44%)	8 (50%)	0.723*
Intravenous antibiotics needing (%)	10 (77%)	10 (77%)	1.000**	12 (75%)	13 (81%)	1.000**	12 (75%)	9 (56%)	0.458**
Mean number (SD) RBC transfusions	0.9 (3.3)	1.6 (2.0)	**0.012°**	0.8 (3.0)	0.7 (1.3)	0.085°	0.8 (3.0)	1.8 (4.9)	**0.024°**
Median number PLT transfusions (range)	2 (0–15)	3 (1–7)	**0.012°**	2 (0–15)	2 (1–4)	0.750°	2 (0–15)	2 (1–10)	0.421°
Median hospitalization duration, days (range)	21 (18–59)	28 (17–48)	**0.022°**	22 (18–59)	23 (6–29)	0.354°	22 (18–59)	22 (18–66)	0.894°

*RBC* red blood cells, *PLT* platelets, *ANC* absolute neutrophilis count, *SD* standard deviation

^*^ Pearson’s chi square test; ** Fisher’s exact test

° Mann–Whitney test

**Table 5:** Post-transplant clinical outcomes by the received G-CSF formulation. Pairwise propensity score matching analysis in myeloma patients

Results	Biosimilar Peg-filgrastim, *N* = 45	Lenograstim, *N* = 45	*p*-value	Biosimilar Peg-filgrastim, *N* = 22	Originator Peg-filgrastim, *N* = 22	*p*-value	Biosimilar Peg-filgrastim, *N* = 57	Biosimilar Filgrastim *N* = 57	*p*-value
Engraftment									
Median days (range) at ANC > 0.5 × 10^9^/L	10 (9 − 12)	12 (9 − 22)	** < 0.001°**	10 (9 − 12)	10 (9 − 10)	**0.015°**	10 (9 − 12)	11 (5 − 15)	** < 0.001°**
Median days (range) PLTs > 20 × 10^9^/L	11 (9 − 15)	15 (10 − 35)	** < 0.001°**	11 (9 − 16)	11 (9 − 18)	0.798°	11 (9 − 16)	12 (5 − 40)	** < 0.001°**
Febrile neutropenia episodes (%)	5 (11%)	5 (11%)	1.000*	0 (%)	1 (4%)	1.000**	5 (9%)	3 (5%)	0.463**
Microbiologically documented infections (%)	14 (31%)	14 (31%)	1.000*	10 (45%)	8 (36%)	0.540*	17 (30%)	19 (33%)	0.687*
Intravenous antibiotics needing (%)	19 (42%)	19 (42%)	1.000*	10 (45%)	9 (41%)	0.761*	22 (39%)	22 (39%)	1.000*
Mean number (SD) RBC transfusions	0.1 (0.4)	0.6 (1.5)	**0.042°**	0.2 (0.5)	0.2 (0.5)	1.000°	0.1 (0.4)	0.4 (0.9)	0.229°
Median number PLT transfusions (range)	1 (0 − 3)	2 (0 − 12)	** < 0.001°**	1 (0 − 5)	1 (0 − 2)	0.960°	1 (0 − 5)	1 (0 − 5)	**0.008°**
Median hospitalization duration, days (range)	18 (14 − 34)	21 (15 − 27)	** < 0.001°**	18 (15 − 24)	18 (15 − 25)	1.000°	18 (14 − 30)	18 (14 − 30)	0.679°

Bold values are those statistically significant

*RBC* red blood cells, *PLT* platelets, *ANC* absolute neutrophilis count, *FUO* fever of unknown origin, *SD* standard deviation

^*^ Pearson’s chi-square test; ** Fisher’s exact test

° Mann–Whitney test

### Costs analysis

The average total cost per patient of biosimilar Peg-filgrastim was € 18218.9 compared to € 23707.8, € 20677.3, and € 19754.9 of Lenograstim, originator Peg-filgrastim and biosimilar Filgrastim, respectively (Table 6). The main driver of the cost resulted to be the hospitalization followed by PLT transfusion and G-CSF treatments. The average cost savings per patient in favor of biosimilar Peg-filgrastim were € 5488.9, € 2458.4, and € 1536.0 for Lenograstim, originator Peg-filgrastim, and biosimilar Filgrastim, respectively (Table 6).

**Table 6:** Cost analysis results

	Biosimilar Peg-filgrastim	Biosimilar Filgrastim	Lenograstim	Originator Peg-filgrastim
G-CSF treatments	390.0 €	511.2 €	791.6 €	1000.0 €
Intravenous antibiotics	145.3 €	163.8 €	194.7 €	163.8 €
RBC transfusions	54.3 €	126.7 €	144.8 €	72.4 €
PLT transfusions	418.0 €	836.0 €	836.0 €	418.0 €
Hospitalization	17211.3 €	18117.2 €	21740.6 €	19023.1 €
***Total***	***18218.9 €***	***19754.9 €***	***23707.8 €***	***20677.3 €***
*Savings*	*-*	*1536.0 €*	*5488.9 €*	*2458.4 €*

*G-CSF* granulocyte colony stimulating factor, *RBC* red blood cells, *PLT* platelets

## Discussion

Our study showed the efficacy and safety of biosimilar Peg-filgrastim in post-transplant engraftment among myeloma and lymphoma patients undergoing ASCT. The median time to neutrophil and platelet engraftment was 10 and 11 days, similarly to that observed in our historical cohorts of patients receiving originator Peg-filgrastim and significantly shorter than those receiving short-acting G-CSFs (i.e., Lenograstim or biosimilar Filgrastim). Pegylated G-CSF was associated with a significantly faster neutrophil engraftment in ASCT in other studies, substantially conducted by using the originator [17–19]. However, several previous reports suggested the comparability for both pharmacokinetic and pharmacodynamic properties between biosimilar Peg-filgrastim and its originator [20]. Indeed, our study confirmed in the setting of autologous transplant the equivalence in terms of clinical efficacy of both biosimilar and originator Peg-filgrastim, being significantly superior to biosimilar Filgrastim and Lenograstim in terms of neutrophil and platelet engraftment. Data about biosimilar Peg-Filgrastim are still scarce in this context. Recently, some studies have been published, all together showing a slightly superiority of biosimilar pegylated formulations over the short-acting G-CSFs in myeloma and lymphoma patients undergoing ASCT [11, 21]. The physiological reason for the better performance of pegylated G-CSF formulation can be found looking at the pharmacokinetics of Peg-filgrastim. Indeed, its pharmacological profile allows the same powerful effect on myeloid progenitors with the advantage of a single and fixed-dose injection given per cycle, thanks to reduced renal clearance and extended half-life [22, 23]; the only pathway of Peg-filgrastim elimination is the neutrophil-mediated clearance [24]. However, in some studies this biological advantage did not translate in a meaningful better time to neutrophil recovery, mainly because several confounding factors basically due to patient’s selection biases and variability were present in those studies, including population age, CD34 + -infused cells, disease stage, and prior exposure to chemotherapy or radiotherapy. Several factors, other than G-CSFs, are indeed known as able to significantly affect the neutrophil and platelet engraftment after ASCT [25]. The most relevant are age at transplant, number of CD34 + -infused cells, disease stage, and previous radiant treatment [25]. In our study, at least two of those factors were able to negatively influence the post-transplant engraftment in biosimilar Peg-Filgrastim patient cohort, since the median age of these patients was significantly higher and they received a significantly lower number of CD34 + . On the contrary, no significant differences were found in terms of previous chemotherapeutic lines and previous radiotherapy. In addition, we carried out a sub-analysis on lymphoma and myeloma patients separately by PSM to generate different pair wise groups with balanced distribution of specific baseline features (age, gender, and number of infused CD34 +). Even in this “unbiased” model, we observed a significant advantage at least in terms of neutrophil engraftment for pegylated G-CSF formulations compared to short-acting ones, confirming the better performance observed in the overall patient population. In our study, we observed a slightly lower incidence of febrile neutropenia episodes and documented infections with consequent lower broad-spectrum antibiotic consumption in patients who received biosimilar Peg-filgrastim; however, it is not statistically significant. This trend is consistent with that reported in the study of Martino et al. [11] and other groups [19, 26–28], where it however reached the statistical significance. Our data about febrile neutropenia episodes and documented infections could be however potentially influenced by the higher number of myeloma patients observed in the biosimilar Peg-filgrastim group. Indeed, taking a look at lymphoma sub-analysis, we can easily understand that the number of febrile episodes and antibiotic needing was quite similar among all the patient’s cohorts, suggesting that febrile neutropenia was more rarely detected in myeloma patients, irrespective to the received G-CSF formulation, in accordance with our previous published data [29]. Further studies are warranted to better clarify whether the G-CSF formulation can potentially influence the occurrence of post-transplant febrile neutropenia, documented infections and antibiotic needing in ASCT. As a consequence of a shorter engraftment time, from our study, we observed a significantly lower transfusion needing and a shorter hospitalization time among patients receiving biosimilar and originator Peg-filgrastim. In particular, this difference was statistically significant compared to Lenograstim patient cohort, and less evident if compared to biosimilar Filgrastim. Even if hospitalization duration could be potentially affected by several confounding factors, this datum seems to be relevant in our opinion, since less hospitalization time means a better management of health resources in terms of both organization and costs. In general, it has already been widely discussed and demonstrated in the literature how the lower cost of biosimilar drugs and the consequent savings derived from their use can lift the financial burden of health care systems and increase patient access to drugs [30–32]. Over the past decade, the biosimilar Filgrastim transformed patient access, with clear evidence of clinical benefits in preventing febrile neutropenia at reduced costs (savings conservatively estimated at 39% in Europe) and in 2019 the licensing in Europe of the biosimilar Peg-filgrastim provided the opportunity to offer the additional benefits of long-acting G-CSF over short-acting G-CSF at a reduced cost [33–35]. Our study showed that potential cost savings per patient range from approximately € 1500 to approximately € 5500 by adopting the biosimilar Peg-filgrastim in place of biosimilar Filgrastim, originator Peg-filgrastim or Lenograstim. These benefits could be more substantial at a population level. For example, the economic impact of introducing biosimilar Peg-filgrastim compared to the current standard G-CSF practice in France was estimated to generate a cost saving from € 51007531 to € 287344835 over 5 years switching from the current standard practice to biosimilar Peg-filgrastim [36]. In Germany, the health-economic impact of biosimilar Peg-filgrastim in the real world for healthcare system would generate a potential annual savings of up to € 56.4 million, with a saving of up to € 4199 per patient compared to originator product [37]. Most economic evaluations of biosimilars consider only the cost of the drug, but it is fundamental in economic evaluations to estimate the real savings beyond the cost of the drug, especially in the case of differences in the form of administration or in adherence, differences in use of healthcare resources, or to consider value-added services. Our study showed that cost savings of biosimilar Peg-filgrastim were in fact mainly attributable at the inpatient management. A real-world data study on primary prophylaxis with Peg-filgrastim vs Filgrastim in cancer patients at intermediate-to-high risk of febrile neutropenia showed that biosimilar Peg-filgrastim was dominant (with a cost saving of $ 5703 and a gain of 0.28 quality-adjusted life year (QALY)) compared with biosimilar Filgrastim for the high-risk group and a cost/QALY of $ 14502 for the intermediate-risk group [38]. In line with our study, the main saving of biosimilar Peg-filgrastim vs biosimilar Filgrastim was primarily driven by a lower cost of inpatient febrile neutropenia management for patients receiving biosimilar Peg-filgrastim [38]. The savings arising from the cost containment using biosimilar Peg-filgrastim could be reallocated to increase patient access to innovative therapies, to move therapy to an earlier line of treatment, to increase the number of healthcare staff thus resulting in a better health outcome for more patients. Peg-filgrastim administration was safe, with no reported grade 3–4 adverse events and the safety profile was similar to that seen for the other G-CSF formulations. At the same time, we observed a superimposable rate of mucositis and diarrhea among the four patient cohorts and the TRM was quite similar, overall ranging between 1 and 4%. This is quite in contrast to some previous report [11, 39] in which the authors showed a lower incidence of mucositis and grade 2–3 diarrhea in patients who received pegylated G-CSFs both in myeloma patients undergoing ASCT and in breast cancer patients, respectively. Multiple factors can potentially explain this difference, keeping in mind that post-transplant gastrointestinal toxicities are usually related to several variables not always easy to predict and control. The present study has some limitations, basically due to the study design, including the use of historical controls. The differences detected in the baseline demographic and clinical characteristics among the four patient cohorts can potentially affect the reliability of our findings. In addition, we analyzed only a small amount of lymphoma patients who received biosimilar Peg-filgrastim after ASCT. Even in this last group, although we used a PSM to generate different pair-wise groups with balanced distribution of specific baseline features in order to maximize the comparability among the different patient cohorts, our results should be read carefully particularly if we consider the limited sample size. In conclusion, being aware of the limitations discussed above, from our study biosimilar Peg-filgrastim seems to be as effective as the originator and more effective than short-acting G-CSF formulations (Lenograstim and biosimilar Filgrastim) in terms of post-transplant engraftment in myeloma and lymphoma patients undergoing ASCT. In addition, pegylated formulations seem to be associated to a better patient clinical management in terms of transfusion needing, febrile neutropenia, and hospitalization duration. Finally, from our pharmacoeconomic evaluation, biosimilar Peg-filgrastim was cost-effective when compared with the other G-CSF formulations and savings derived from its use may contribute to an expansion of medical treatment options for patients, hence concomitantly contributing to the long-term sustainability of the healthcare system. We believe that our findings could help clinicians and healthcare decision-makers in the better management of febrile neutropenia prophylaxis of myeloma and lymphoma patients undergoing ASCT.

## Data Availability

The datasets are available from the corresponding author on reasonable request.
